# Circulating Level of Neutrophil Extracellular Traps Is Not a Useful Biomarker for Assessing Disease Activity in Antineutrophil Cytoplasmic Antibody-Associated Vasculitis

**DOI:** 10.1371/journal.pone.0148197

**Published:** 2016-02-03

**Authors:** Huan Wang, Li-Li Sha, Tian-Tian Ma, Lu-Xia Zhang, Min Chen, Ming-Hui Zhao

**Affiliations:** 1 Renal Division, Department of Medicine, Peking University, First Hospital, Beijing 100034, China; 2 Peking University Institute of Nephrology, Beijing 100034, China; 3 Key Laboratory of Renal Disease, Ministry of Health of China, Beijing 100034, China; 4 Key Laboratory of Chronic Kidney Disease Prevention and Treatment (Peking University), Ministry of Education, Beijing 100034, China; 5 Peking-Tsinghua Center for Life Sciences, Beijing 100034, China; 6 Guizhou Medical University, Guiyang 550004, China; The Hospital for Sick Children and The University of Toronto, CANADA

## Abstract

Antineutrophil cytoplasmic antibody (ANCA)-associated vasculitis (AAV) is a group of life-threatening disorders, and frequently affects the kidneys. This study investigated whether the circulating neutrophil extracellular traps (NETs) levels were associated with disease activity of AAV. We collected serum samples from 34 patients with AAV in active stage and 62 patients with AAV in remission. Cell free DNA in serum was quantified using the Quant-iT PicoGreen assay. NETs associated MPO-DNA complexes, citrullinated-histone H3-DNA (cit-H3-DNA) complexes and the concentration of deoxyribonuclease I (DNase I) were quantified using ELISA. The activity of DNase I was quantified using radial enzyme-diffusion method. Associations between circulating levels of NETs with clinico-pathological parameters were analyzed. Serum levels of NETs in active AAV patients were significantly higher than those in healthy controls, and the level of cell free DNA correlated with C-reactive protein (CRP). However, no correlation was found between MPO-DNA complexes or cit-H3-DNA complexes level and CRP. Also there was no significant correlation between NETs level and initial serum creatinine, estimated glomerular filtration rate (eGFR), crescents formation or Birmingham Vasculitis Activity Score (BVAS). Furthermore, there was no significant difference of serum levels of cell free DNA or MPO-DNA complexes between active stage and remission of AAV. In conclusion, circulating levels of NETs cannot be used as a biomarker to assess disease activity in AAV patients.

## Introduction

Antineutrophil cytoplasmic antibody (ANCA)-associated vasculitis (AAV) is a group of life-threatening disorders, including microscopic polyangiitis (MPA), granulomatosis with polyangiitis (GPA) and eosinophilic granulomatosis with polyangiitis (EGPA) [1]. As the serological hallmarks for AAV, ANCAs are autoantibodies predominantly directed against neutrophil cytoplasmic constituents, in particular, myeloperoxidase (MPO) and proteinase 3 (PR3).

In 2004, Brinkmann *et al*. [2] found that activated neutrophils could release chromatin and granule proteins that together form extracellular fibers which bind Gram-positive and -negative bacteria. These neutrophil extracellular traps (NETs) are composed of decondensed extracellular chromosomal DNA decorated with neutrophil-derived granule proteins which could dismantle and kill bacteria extracellularly [2]. Recently, NETs have been discovered as a novel role of neutrophils in the pathogenesis of AAV [3]. Kessenbrock *et al*. [3] discovered that ANCA-stimulated neutrophils could induce the formation of NETs, which contain MPO and PR3. The deposition of NETs was found in inflamed skin vessels [4], glomeruli [3, 5] and thrombosis [5, 6]. In a murine model of autoimmune vasculitis, released NETs could activate bone marrow-derived dendritic cells and transfer neutrophil-derived PR3 and MPO molecules to these cells [7]. A recent study [8] assessed the formation and regulation of NETs in AAV patients. They evidenced the high ability for NETs induction and low ability for NETs degradation of serum in patients with AAV.

In systemic lupus erythematosus (SLE), decreased degradation of NETs is associated with disease activity [9], and impairment of deoxyribonuclease I (DNase I) function correlated with kidney involvement [10]. In thrombotic microangiopathies (TMA), the DNA-histone complexes which were released during acute TMA correlate with disease activity during therapy, and the amount of circulating DNA and MPO was inversely correlated with the number of circulating platelets [11]. The level of NETs is also a predictive marker for the occurrence of TMA after allogeneic stem cell transplantation [12]. Intuitively, we considered that the circulating NETs might be associated with disease activity in AAV patients. In this study, we tested the circulating levels of NETs, including MPO-DNA complexes, citrullinated-histone H3-DNA (cit-H3-DNA) complexes and cell free DNA, which are important components of NETs, in AAV patients in both active stage and remission, and correlated them with clinical and pathological parameters.

## Materials and Methods

### 2.1 Patients

Blood samples of 34 active AAV at initial onset and before commencing immunosuppressive therapy, diagnosed at Peking University First Hospital from August 2013 to June 2014, were collected in this study. The authors had access to identifying information during and after data collection. All the patients met the Chapel Hill Consensus Conference (CHCC) definition of AAV [1]. Patients with secondary vasculitis or with co-morbid renal diseases, such as anti-GBM nephritis, IgA nephropathy, diabetic nephropathy, lupus nephritis or membranous nephropathy, were excluded. The disease activity of AAV was assessed according to the Birmingham Vasculitis Activity Score (BVAS) [13]. Nineteen of the 34 patients received renal biopsy at diagnosis. Serum samples of 62 patients with AAV, who achieved complete remission after immunosuppressive therapy, were also collected at their regular ambulatory visits. Remission was defined as the ‘absence of disease activity attributable to active disease qualified by the need for ongoing stable maintenance immunosuppressive therapy’ (complete remission), or ‘at least 50% reduction of the disease activity score and absence of new manifestations’ (partial remission), as described previously [14]. Estimated glomerular filtration rate (eGFR) was calculated using the modification of diet in renal disease equations [15]. All the AAV patients recruited in the study were MPO-ANCA positive, since PR3-ANCA positive patients only constitute a minority of AAV patients in China, as shown in our previous studies [16–18]. Among the above-mentioned AAV patients, there were 20 patients with serum samples both in active stage and remission. Serum samples of 22 healthy blood donors were collected as the healthy controls. Among of these healthy controls, 10 were male, 12 were female, with an age of 32.3±7.2 years. Venous blood was collected into red cap vacuum blood collection tubes without anticoagulants. After collection of the whole blood, allowing the blood to clot by leaving it undisturbed at room temperature for 1h, and then blood sample was centrifuged at 1000g for 10 min at 4°C. Serum was then obtained and kept at -80°C until use. This research was approved by the ethics committee of the Peking University First Hospital and was in compliance with the Declaration of Helsinki. Written informed consent has been obtained from each participant.

### 2.2 Detection of serum ANCA

All the sera were tested for ANCAs by both indirect immunofluorescence (IIF) assay and antigen-specific enzyme-linked immunosorbent assay (ELISA), according to the manufacturer’s instructions (EUROIMMUN).

### 2.3 Renal histology

Renal histology of patients with AAV was evaluated according to the previous standardized protocol [19–21]. The presence of glomerular lesions, including fibrinoid necrosis, crescents, and glomerulosclerosis, was calculated as the percentage of the total number of glomeruli in biopsy findings. Interstitial and tubular lesions were scored semiquantitatively on the basis of the percentage of the tubulointerstitial compartment that was affected: interstitial infiltrate (“-”for 0%, “+” for 0–20%, “++” for 20–50% and “+++” for >50%), interstitial fibrosis (“-”for 0%, “+” for 0–50% and “++”for >50%) and tubular atrophy (“-”for 0%, “+” for 0–50% and “++”for >50%).

### 2.4 Evaluation of serum NETs level

NETs are composed of a DNA backbone decorated with granule proteins such as MPO, cit-H3 and elastase. NETs are also the major source of circulating cell free DNA. To identify NETs, we quantified serum NETs level by detecting the major NETs components, cell free DNA, MPO-DNA complexes and cit-H3-DNA complexes in serum samples, which is consistent with most previous studies [3, 10, 12, 22, 23].

As an indirect way to assess NETs, cell free DNA in serum was quantified using Quant-iT PicoGreen DNA quantification kit (Invitrogen) in accordance with the manufacturer’s instructions. Briefly, the DNA standard curve (from 1 ng/mL to 1000ng/mL) were diluted and then incubated for 5 minutes with Quant-iT PicoGreen reagent at room temperature before measuring fluorescence. Samples were compared to the standard curve and the results expressed in ng/mL. Fluorescence signals were measured in a microplate fluorescence reader (TristarTM LB941) with filter settings of 485 nm (excitation) and 538 nm (emission).

NETs associated MPO-DNA complexes were quantified as previously described [3, 23]. Considering there was no MPO-DNA complexes standard, we fixed one patient’s sera as the ‘standard’ to control for variability. The NETs level of ‘standard’ was relatively high, then it also could be used as the positive control. In brief, 5μg/ml of mouse anti-human MPO antibody (ABD Serotec) was coated to 96-well microtiter plates. After blocking with 1% BSA, serum sample was added together with a peroxidase-labeled anti-DNA monoclonal antibody (component 2 of the Cell Death ELISA kit, Roche). After incubation, the peroxidase substrate was added according to the manufacturer's instructions. The optical absorbance was measured at 405 nm in an ELISA reader (Bio-Rad 550; Bio-Rad Laboratories, Tokyo, Japan).

Among the above-mentioned patients, we recruited 22 patients in active stage with sufficient serum samples, and randomly selected 30 patients in remission, to detect the cit-H3-DNA complexes based on its assocation with NETs as the method of quantifying MPO-DNA complexes mentioned above. Among them, 10 patients who had blood samples both in active stage and remission were included. Also we employed one patient’s sera as the ‘standard’ to control for variability, as mentioned above. The antibody (5μg/ml) used to coat the 96-well plate was anti-histone H3 (citrulline) antibody (ab80256, Abcam). 1% BSA was used for blocking. Then serum sample together with the peroxidase-labeled anti-DNA monoclonal antibody (Roche) was added. After that, the peroxidase substrate was added according to the manufacturer's instructions. The optical absorbance was measured in an ELISA reader (Bio-Rad 550; Bio-Rad Laboratories, Tokyo, Japan) at 405 nm.

### 2.5 Evaluation of renal NETs level

The renal NETs level was measured using immunohistochemistry. Immunohistochemical staining for cit-H3 was performed on the paraffin-embedded tissues (10 active AAV patients and 4 normal controls). Endogenous peroxidase was blocked with 3% H_2_O_2_ in distilled water for 30 min at 37°C. All the sections were incubated by microwave oven in EDTA buffer (pH 9.0) for antigen retrieval. After blocking with 3% BSA at 37°C, tissue slides were incubated with rabbit polyclonal antibody against human to citrulline histone H3 (ab5103, Abcam) at 4°C overnight, and then diaminobenzidine staining was performed. The nuclei were stained with hematoxylin. The result of immunohistochemistry was evaluated (at 20× and 40× magnification) by two independent investigators (pathologists) who were blinded to patients’ clinical data.

### 2.6 Evaluation serum DNase I level and DNase I activity

The concentration of serum DNase I was measured using a commercial ELISA kit according to the manufacturer’s instructions (EIAab, Catalog No: E1127h, Wuhan, China). The optical absorbance was measured at 450 nm in an ELISA reader (Bio-Rad 550; Bio-Rad Laboratories, Tokyo, Japan). Samples were compared to the standard curve and the results were expressed in ng/mL.

Among the above-mentioned patients, we recruited 22 patients in active stage with sufficient serum samples, and randomly selected 30 patients in remission, to detect the DNase I activity. DNase I activity was measured using radial enzyme-diffusion method, as previously described [24]. To 12ml of 10mM Tris/Ca/Mg buffer, 1.2ml of DNA from calf thymus (D4522, sigma) at 5mg/ml, 3μl ethidium bromide (EB) and 13.2ml melted 2% agarose (Biowest), were added. Then the substrate was poured onto the gel plate. Wells were cut of 1mm diameter, filled with 2μl standards and serum sample, then the plate was incubated at 37°C overnight. The plate was photographed on a UV Transilluminator. The area of dark circles of hydrolysed DNA was scanned by Image-Pro Plus 6.0.

### 2.7 Statistical analysis

Data were expressed as mean±SD (for data that were normally distributed) or median and interquartile range (IQR; for data that were not normally distributed). Differences of quantitative parameters between two groups were assessed using the t-test (for data that were normally distributed) or the nonparametric test (for data that were not normally distributed). Differences of quantitative parameters more than two groups were assessed using one-way ANOVA (parametric data), or Kruskal-Wallis (non-parametric data) test and followed by post-hoc test. Differences of data that were not normally distributed paired samples were tested using Wilcoxon’s signed rank test. Pearson’s test or Spearman’s test was used for correlation analysis as appropriate. Differences were considered significant if the P-value was <0.05. All the statistics were analyzed using SPSS statistical software (version 13.0, Chicago, Ill, USA).

## Results

### 3.1 General data of the patients

Among the 34 active AAV patients, 18 were male and 16 were female, with an age of 61.97±12.30 years at diagnosis. All these patients were perinuclear ANCA (pANCA) positive and MPO-ANCA positive. The level of initial serum creatinine was 397.34±261.96 μmol/l. The level of BVAS in the 34 patients in the active stage was 19.9±6.1, and in the 62 patients in remission, it was 0. The general data for these patients were listed in Table 1.

**Table 1:** General data of patients with AAV^*^.

Data	Active stage	Remission
Subject number	34	62
Gender (male/female)	18/16	26/36
Age, mean±SD, yr	61.97±12.30	64.74±12.24
MPO-ANCA	34	62
Serum creatinine, mean±SD, μmol /L	397.34±261.96	180.74±140.93
Estimated GFR, median(IQR), ml/minute/1.73 m^2^	24.30±22.60	46.57±26.38
Urinary protein, median(IQR), g/24 hours	0.81 (0.58–1.55)	0.62±0.41
BVAS, mean±SD	19.9±6.1	0
Glomeruli with crescents, mean±SD, %	60.6±19.2 (n = 16)	-

* Values are the number (percentage) unless indicated otherwise. Data were collected at presentation.

AAV = antineutrophil cytoplasmic antibody associated vasculitis; GFR = glomerular filtration rate; BVAS = Birmingham Vasculitis Activity Score.

### 3.2 The levels of NETs

**Fig 1:**
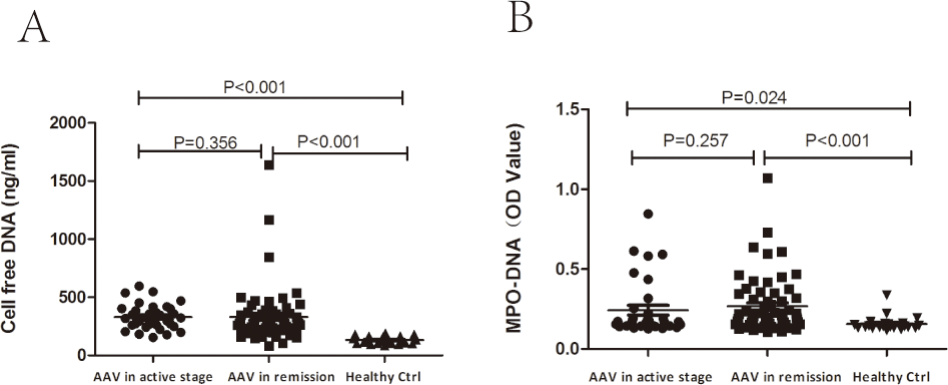
Serum levels of NETs in AAV patients in active stage and remission. A. Quantification of cell free DNA using Quant-iT PicoGreen assay in serum samples from healthy donors (n = 22), individuals with AAV in remission (n = 62) and with active disease (n = 34). B. Quantification of NETs in the serum samples by MPO-DNA complex ELISA. The mean optical density as measured by capture ELISA.

We detected the levels of serum cell free DNA using Quant-iT PicoGreen assay. According to previous studies [12, 25, 26], NETs were the major source of the serum DNA, and the PicoGreen assay has been proved to be a powerful time- and labor-saving tool for evaluating serum NETs in a clinical setting. Serum levels of cell free DNA in patients with AAV in active stage were significantly higher than those in healthy controls [320.10 (258.61, 401.13) ng/ml vs 127.39 (105.56, 161.03) ng/ml, *P*<0.001], while the difference of cell free DNA levels between AAV patients in active stage and in remission was not significant [320.10 (258.61, 401.13) ng/ml vs 276.97 (221.22, 358.39) ng/ml, *P* = 0.356] (Fig 1A).

The levels of MPO-DNA complexes (expressed by the optical density value) in active AAV patients were significantly higher than those in healthy controls [0.17 (0.14, 0.23) vs. 0.14 (0.13, 0.16), *P* = 0.024]. There was no significant difference of serum levels of MPO-DNA complexes between AAV patients in active stage and in remission [0.17 (0.14, 0.23) vs 0.20 (0.16, 0.33), *P* = 0.257] (Fig 1B).

The levels of cit-H3-DNA complexes (expressed by the optical density value) in active AAV patients were significantly higher than the healthy controls [0.19±0.06 vs 0.10±0.01, P<0.001]. Also the cit-H3-DNA complexes levels of patients in active stage was higher than the patients’ level of cit-H3-DNA complexes in remission [0.19±0.06 vs. 0.13±0.03, *P*<0.001].

**Fig 2:**
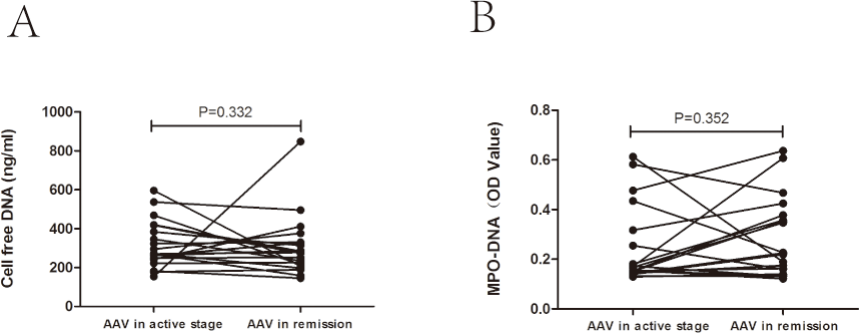
Serum levels of NETs in 20 AAV patients with sequential serum samples. A. Serum levels of cell free DNA. B. Serum levels of MPO-DNA complexes.

We then compared serum levels of cell free DNA and MPO-DNA complexes in 20 AAV patients with serum samples of both active stage and remission. There was still no significant difference of serum cell free DNA between the active stage and remission [271.38 (244.25, 409.22) ng/ml vs. 275.50 (209.34, 329.94) ng/ml, *P* = 0.332], which was consistent with the above-mentioned results. Only 10 of these 20 patients had a decrease in serum level of cell free DNA in remission compared with those in active stage (Fig 2A). We got the similar results regarding the level of MPO-DNA complexes, which didn’t decrease significantly in AAV patients in remission, as compared with the active stage [0.28±0.16 vs. 0.24±0.16, *P* = 0.352]. Only 7 of these 20 patients had a decrease in serum level of MPO-DNA complexes in remission compared with those in active stage (Fig 2B). However, when comparing patients who had a decrease in serum level of cell free DNA or MPO-DNA complexes with those who had an increase in serum level of cell free DNA or MPO-DNA complexes, we failed to find any difference between these two subgroups of patients, including demographic data, medication, or DNase I activity. Then we compared serum level of cit-H3-DNA complexes in AAV patients with serum samples of both active stage and remission. There was still no significant difference between the level of cit-H3-DNA complexes in active stage and remission (0.18±0.04 vs 0.15±0.03, *P* = 0.093).

The serum NETs levels from the MPO-DNA ELISA and the cell free DNA PicoGreen assay were positively correlated (*r* = 0.474, *P* = 0.005); while no significant correlation was found between the level of cit-H3-DNA complexes and the level of MPO-DNA complexes (*r* = -0.008, *P* = 0.972), or the level of cell free DNA (*r* = 0.175, *P* = 0.437).

**Fig 3:**
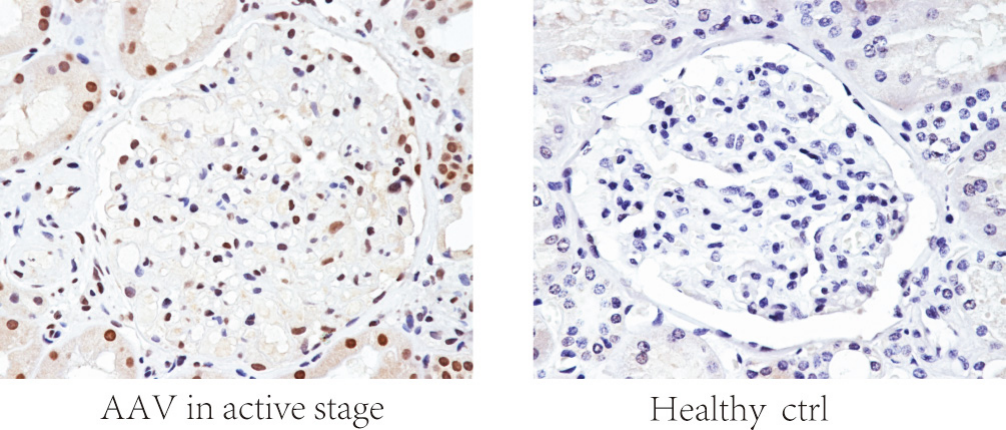
Renal NETs level of AAV patients and healthy control.

Furthermore, we detected NETs level by measuring cit-H3-DNA complexes in renal specimens. The result showed that the level of cit-H3-DNA complexes in active patients was significant higher than healthy controls (0.48±0.06 vs. 0.03±0.04, *P*<0.001) (Fig 3). Correlation analysis showed that there was significant association between the level of cit-H3-DNA complexes in serum and in renal of AAV patients (*r* = 0.706, *P* = 0.023).

### 3.3 Association between serum NETs levels and clinicopathological parameters in AAV patients

Among the 34 active AAV patients, correlation analysis showed that serum levels of cell free DNA weakly correlated with C-reactive protein (CRP) (*r* = 0.396, *P* = 0.027), while MPO-DNA complexes and cit-H3-DNA complexes were not significantly associated with CRP (*r* = 0.338, *P* = 0.063; *r* = -0.106, *P* = 0.655). Also there was no significant correlation between the NETs level and initial eGFR (*r* = 0.155, *P* = 0.382; *r* = 0.195, *P* = 0.269; *r* = -0.106, *P* = 0.639), or serum creatinine (*r* = -0.206, *P* = 0.242; *r* = -0.104, *P* = 0.559; *r* = -0.033, *P* = 0.883), or BVAS (*r* = 0.304, *P* = 0.081; *r* = 0.147, *P* = 0.407; *r* = -0.062, *P* = 0.783). No significant association was found between serum NETs level and the pathology parameters in renal biopsies, including the proportion of crescents formation and fibrinoid necrosis.

### 3.4 Serum DNase I concentration and DNase I activity

**Fig 4:**
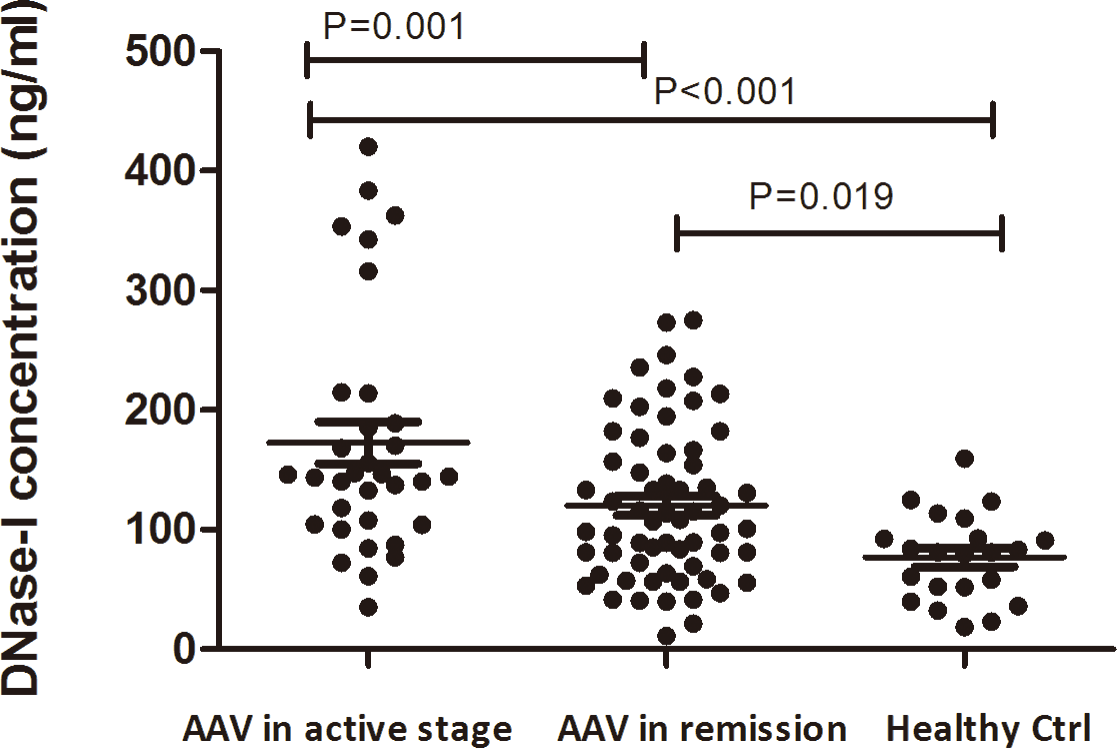
Circulating DNase I concentration in AAV patients.

The concentration of DNase I in AAV patients, which could selectively digest the DNA threads of NETs has been quantified. The serum levels of DNase I were significantly higher in active AAV patients compared with remission patients and normal controls (172.85±100.70 vs. 120.03±64.81 ng/ml, *P* = 0.001; 172.85±100.70 vs. 76.71±36.31 ng/ml, *P*<0.001, respectively) (Fig 4). There was no significant difference of the DNase I activity between the patients in active stage and remission (0.22±0.11 vs 0.21±0.62 U/ml, *P* = 0.857). Correlation analysis showed that the level of cell free DNA was negatively associated with DNase I activity (*r* = -0.499, *P* = 0.021). While no significant correlation was detected between DNase I activity and the MPO-DNA complexes level (*r* = -0.024, *P* = 0.916), or cit-H3-DNA complexes level (*r* = -0.015, *P* = 0.948). No significant association was found between the DNase I concentration and DNase I activity (*r* = -0.139, *P* = 0.559), or BVAS (*r* = -0.017, *P* = 0.925), or CRP (*r* = 0.052, *P* = 0.787).

## Discussion

Neutrophil extracellular traps (NETs) are released by activated neutrophils in response to various stimuli, undergoing a process called NETosis. Recent studies reported that the formation of NETs triggers vasculitis and promotes the autoimmune response against neutrophil components in AAV patients [3]. Nakazawa *et al*. found that the NET induction ability of ANCA positive IgG is correlated with disease activity [8]. A recent study by Fuchs *et al*. found that the levels of plasma DNA were normalized in clinical remission, whereas MPO tended to normalize but remained slightly above control values, indicating that DNA and MPO might reflect the disease activity in TTP patients [11]. The level of circulating cell free DNA is also a predictive marker for the occurrence of TMA after allogeneic stem cell transplantation [12]. Zhang *et al*. found that in SLE, the cell free DNA concentration was positively correlated with the urinary protein, and inversely correlate with the endogenous creatinine clearance rate [27]. In this study, we investigated the association between serum NETs levels and disease activity of AAV.

In our study, we found that the serum levels of NETs in active AAV patients were significantly higher than those in healthy controls. However, it is intriguing that in our study, no significant difference was found in serum levels of cell free DNA and MPO-DNA complexes between AAV patients in active stage and in remission. Although the level of cit-H3-DNA complexes in active patients was higher than the patients in remission, no significant correlation was found between levels of cit-H3-DNA complexes and eGFR, CRP, serum creatinine, or BVAS. Recently, Surmiak *et al*. demonstrated that the 79 bp mitochondrial DNA correlated with active stage of GPA [28]. Another recent study found that the active AAV patients have increased levels of NET remnants in the circulation, and negatively correlated with the levels of PR3-ANCA, but not MPO-ANCA [29]. However, the sample size of PR3-ANCA-positive patients was relatively small in our cohort [16–18], this subgroup of patients were not included. We further tested the concentration and activity of DNase I in AAV patients, which could selectively digest the DNA threads of NETs. We found that the concentration of DNase I in active stage was significantly higher than that in remission, while there was no significant difference of DNase I activity between these two stages. No significant association was found between the concentration of DNase I or DNase I activity and the disease activity. Also there was no significant correlation between the DNase I activity and the MPO-DNA complexes level, or cit-H3-DNA complexes level. It indicated that the level of NETs may not just be influenced by the activity and concentration of DNase I.

On the other hand, although NETs are well known for their proinflammatory role in autoimmune disease, evidence has emerged that NETs also participate in damping inflammation and facilitating tissue repair [30–33]. For example, in a murine model of gout, aggregated NETs promote the resolution of neutrophil-induced inflammation by trapping and degrading proinflammatory mediators [30]. Whether the high levels of NETs in remission might also be involved in regulating inflammation in AAV, exerting the anti-inflammatory effect, remains further investigation.

In conclusion, circulating levels of NETs cannot be used as a biomarker to assess disease activity in AAV patients.
